# Brain tissue oxygen amperometry in behaving rats demonstrates functional dissociation of dorsal and ventral hippocampus during spatial processing and anxiety

**DOI:** 10.1111/j.1460-9568.2010.07497.x

**Published:** 2011-01

**Authors:** Stephen B McHugh, Marianne Fillenz, John P Lowry, J Nicolas P Rawlins, David M Bannerman

**Affiliations:** 1 Department of Psychology, University of OxfordOxford, South Parks Road, Oxford OX1 3UD, UK; 2 Department of Physiology, Anatomy and Genetics, University of OxfordOxford, UK; 3 Department of Chemistry, National University of IrelandMaynooth Co. Kildare, Ireland

**Keywords:** constant potential amperometry, dorsal hippocampus, oxygen, tissue oxygen voltammetry, ventral hippocampus

## Abstract

Traditionally, the function of the hippocampus (HPC) has been viewed in unitary terms, but there is growing evidence that the HPC is functionally differentiated along its septotemporal axis. Lesion studies in rodents and functional brain imaging in humans suggest a preferential role for the septal HPC in spatial learning and a preferential role for the temporal HPC in anxiety. To better enable cross-species comparison, we present an *in vivo* amperometric technique that measures changes in brain tissue oxygen at high temporal resolution in freely-moving rats. We recorded simultaneously from the dorsal (septal; dHPC) and ventral (temporal; vHPC) HPC during two anxiety tasks and two spatial tasks on the radial maze. We found a double-dissociation of function in the HPC, with increased vHPC signals during anxiety and increased dHPC signals during spatial processing. In addition, dHPC signals were modulated by spatial memory demands. These results add a new dimension to the growing consensus for a differentiation of HPC function, and highlight tissue oxygen amperometry as a valuable tool to aid translation between animal and human research.

## Introduction

Techniques that aid the translation between animal and human research are essential to neuroscience. Most of our knowledge of the cellular and molecular mechanisms that underlie behaviour comes from animal experiments that for ethical and practical reasons are impossible in humans. In parallel, non-invasive techniques such as functional magnetic resonance imaging (fMRI) have revolutionized the study of human cognition, but cannot be used to study behaviour in animals because the subject must remain motionless. Moreover, fMRI uses an oxygen-based signal that is difficult to compare directly with the measures of neuronal activity typically recorded in animals. Consequently, there is a need for techniques that help to bridge this gap.

With this in mind, here we used constant potential amperometry (CPA), an *in vivo* amperometric technique with sub-second temporal resolution, to measure region-specific changes in brain tissue oxygen (O_2_) in freely-moving rats. Several laboratories have used oxygen CPA to investigate the neural basis of the blood oxygen level-dependent (BOLD) signal (Thompson *et al.*, 2003; Offenhauser *et al.*, 2005; Masamoto *et al.*, 2008). These and other studies have shown that increases in both tissue O_2_ and BOLD are dependent upon increased cerebral blood flow (CBF), and that increased CBF occurs in response to increased excitatory synaptic activity (Lauritzen, 2005). Recently, we have shown close concordance between CPA-measured tissue O_2_ and the BOLD signal when recorded simultaneously in the MRI scanner (Lowry *et al.*, 2010). However, these experiments were all performed in anaesthetized animals, and the primary, and as yet unexploited, advantage of CPA is that it can be used in freely-moving animals to study region-specific activation during behaviour. Here we used CPA to investigate differentiation of function along the septotemporal axis of the hippocampus (HPC).

Several lines of evidence suggest that the septal HPC [dorsal HPC (dHPC) in rodents, posterior HPC in primates] plays a preferential role in spatial memory, whereas the temporal HPC [ventral HPC (vHPC) in rodents, anterior HPC in primates] plays a preferential role in anxiety (Bannerman *et al.*, 2004). In rats, dHPC but not vHPC lesions impair spatial memory (Moser *et al.*, 1995; Bannerman *et al.*, 1999), whereas vHPC but not dHPC lesions reduce anxiety (Bannerman *et al.*, 2002; Kjelstrup *et al.*, 2002; McHugh *et al.*, 2004). However, in these experiments we are studying dysfunctional rather than functional brains. In humans, functional neuroimaging studies report greater activation of posterior HPC during spatial navigational tasks (Maguire *et al.*, 1997; Hartley *et al.*, 2003), but greater activation of anterior HPC during anxiety (Hasler *et al.*, 2007), consistent with the rodent lesion data. However, the behavioural tasks used and the methodologies employed differ markedly, as well as the species in question. To this end, here we measured dHPC and vHPC tissue O_2_ signals during tests of unconditioned anxiety and spatial processing in freely-moving rats.

Anxiety was investigated using two unconditioned ethological tests: the black/white alley and food neophobia. Although often conflated, fear and anxiety are dissociable psychological constructs, subserved by different brain circuits. Fear is characterized by phasic responses to explicit and often conditioned cues, and is associated with imminent or present danger; whereas anxiety is associated with potential danger and is characterized by tonic responses to situations of uncertainty or conflict (Davis & Shi, 1999; Gray & McNaughton, 2000; Bannerman *et al.*, 2004). Thus, in psychological terms, anxiety is defined as the response to the conflict (e.g. approach/avoid) between competing goals, and is not a response to the specific danger or threat cues themselves (Gray & McNaughton, 2000). For example, in the black/white alley, this manifests as an approach/avoid conflict between rats’ natural preference for dark, enclosed spaces vs. their instinctive drive to explore novel environments. In the food neophobia test, prior food deprivation produces a conflict between hunger and rats’ innate caution of eating in novel environments and/or novel foodstuffs. These tests have previously been validated with benzodiazepines (Shephard & Estall, 1984; McHugh *et al.*, 2004) and, importantly, both are sensitive to vHPC but not dHPC lesions (Bannerman *et al.*, 2002; McHugh *et al.*, 2004). We predicted these tests would elicit higher tissue O_2_ signals in vHPC than dHPC.

Spatial processing was investigated using two radial maze tasks. In the eight-arm spatial working memory task, all eight arms of the maze were baited at the start of each trial and the rat was required to visit each arm only once per trial for optimum performance (i.e. win-shift behaviour). In the one-arm control task, rats repeatedly entered the same arm of the maze for food reward. Thus, the sensorimotor (e.g. physical activity) and motivational demands of the two tasks were the same, but only the eight-arm task required spatial working memory. A previous study has shown that the eight-arm task elicits greater HPC immediate-early gene (IEG) expression than the one-arm task (Vann *et al.*, 2000). Moreover, spatial working memory is sensitive to dHPC but not vHPC lesions (Pothuizen *et al.*, 2004). Hence, we predicted that spatial processing would elicit higher tissue O_2_ signals in dHPC than vHPC, and also that the eight-arm task would elicit higher signals than the one-arm task.

Finally, we investigated the extent to which dHPC tissue O_2_ signals were dependent upon physical activity by simultaneously recording tissue O_2_ and local field potentials (LFPs) during sleep and waking.

## Materials and methods

### Subjects

This study used 13 naïve male Sprague–Dawley rats (Harlan, Bicester, UK). They were approximately 2 months old at the start of testing, and were housed individually in a temperature- and humidity-controlled room under a 12 h light : dark cycle (lights on 07:00–19:00 h). Testing was carried out during the light phase. Rats had free access to food and water, except prior to the food neophobia test when access to food was removed for approximately 16 h, and during radial maze training when they were maintained on a restricted diet at approximately 85% of free-feeding weight. The experiments were conducted in accordance with the United Kingdom Animals Scientific Procedures Act (1986) under project licence PPL 30/1989.

### Electrode construction

Carbon paste electrodes (CPEs) were constructed from 50-mm-long 8T (200 μm bare diameter, 270 μm coated diameter) Teflon®-coated silver wire (Advent Research Materials, Suffolk, UK) soldered into gold-plated socket contacts (E363/0, Plastics One, Roanoke, VA, USA). The Teflon® insulation was slid along the wire to create a 2-mm-deep cavity that was packed with carbon paste using a bare silver wire as a plunger. Carbon paste was prepared by thoroughly mixing 2.83 g of carbon powder (UCP-1-M, Ultra Carbon Bay City, MI, USA) and 1.0 mL of silicone oil (Sigma-Aldrich, Catalogue No. 17,563-3), as previously described (O’ Neill *et al.*, 1982). LFP, reference and auxiliary electrodes were made from 50-mm-long 5T Teflon®-coated silver wire (125 μm bare diameter, 177 μm coated diameter), with one end soldered into a gold-plated contact (E363/0, Plastics One). Dual CPE/LFP electrodes were made by twisting the CPE and LFP electrode around each other and cutting the end of the LFP electrode with a scalpel so that the active tips were level in the dorsal-ventral plane.

### Surgery

Eight rats were implanted with CPEs into the dHPC of one hemisphere and the vHPC of the other hemisphere (right dHPC/left vHPC *n*=4; left dHPC/right vHPC *n*=4), and one rat was implanted with CPEs into the dHPC of both hemispheres. Four additional rats were implanted with CPE and LFP electrodes into the dHPC (bilateral, *n*=1; unilateral dual electrode, *n*=3). The rats were anaesthetized with Avertin (290 mg/kg, i.p.; *n*=8) or Isoflurane (3–4% in 4 L/min O_2_ for induction, 1.5–2% in 1 L/min O_2_ for maintenance; *n*=5), and placed in a stereotaxic frame with the head level between bregma and lambda. An incision was made along the midline and the periosteum resected. Skull holes were drilled to allow the insertion of electrodes and jewellers’ screws. Auxiliary electrodes were wrapped around a skull screw and reference electrodes were implanted into the cortex. CPEs were implanted at the following coordinates: A–P: −3.6, M–L: ± 2.2, D–V: −3.2 (dHPC); A–P: −5.3, M–L: ± 4.7, D–V: −6.5 (vHPC). Rats with both CPE and LFP electrodes had separate reference and auxiliary electrodes for each circuit. The electrodes were secured with dental cement (‘Simplex Rapid’, Associated Dental Products, Swindon, UK), and the gold contacts from each electrode were inserted into a six-channel electrode pedestal (MS363, Plastics One), which was secured to the skull with dental cement. Analgesia was given in the form of either carprofen (4 mg/kg; s.c.) or meloxicam (2 mg/kg; s.c.).

### Amperometric techniques

Changes in tissue oxygen were recorded using CPA at CPEs, as described previously (Lowry *et al.*, 1997; Bazzu *et al.*, 2009). Briefly, a constant potential (−650 mV) was applied to the CPE using a custom-designed low-noise potentiostat (Electrochemical and Medical Systems, Newbury, UK). This potential is in the diffusion-limited region of the reduction wave for oxygen. When this potential is applied, the electrochemical reduction of dissolved O_2_ occurs on the surface of the CPE such that changes in the local tissue O_2_ concentration produce directly proportional changes in the measured current (Hitchman, 1978). The reduction of O_2_ at carbon electrodes is a two-electron process producing H_2_O_2_: 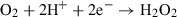

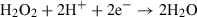
 Because H_2_O_2_ is poorly oxidized at carbon, irreversible electrochemical behaviour is observed. The rate-limiting step for this reduction is the initial one-electron step followed by protonation of the superoxide ion and further reduction (Taylor & Humffray, 1975; Zimmerman & Wightman, 1991).

### Data recording

Rats were connected to the potentiostat via a six-channel commutator (SL6C, Plastics One) and head-stage pre-amplifier using screened cables (363-363 6TCM, Plastics One). A Powerlab® 4/20 (AD Instruments, Oxon, UK) was used for analogue/digital conversion, and data were collected on a Windows PC running Chart® v4 software (AD Instruments). Tissue O_2_ data were sampled at 10 Hz and smoothed with a nine-point Bartlett triangular window.

### Signal stability

Several steps were taken to promote tissue O_2_ signal stability *in vivo*. First, recordings began at least 7 days after surgery to allow any damaged tissue to recover. Second, prior to any experiments, the potential was applied to the electrodes for several hours *in vivo* (minimum 8 h, typically 24 h; one to three sessions) to allow the electrodes to condition. Previous evidence suggests that the response properties of oxygen electrodes are more variable in the first few hours after the potential is first applied, but that the signal stabilizes after conditioning (Vanderkooi *et al.*, 1991). One hypothesis for this stabilizing effect is that, during conditioning, oil is removed from the surface of the CPE, which makes the surface more powder-like and increases the rate of charge transfer (Ormonde & O'Neill, 1989; O’ Neill, 1993; Kane & O’ Neill, 1998). Third, at the start of each experimental session the potential was applied to the electrodes for at least 30 min before any experiments began to allow the signal to settle to a stable baseline. Whenever a polarizing potential is applied to a CPE, even after electrode conditioning, a large capacitance current is observed that typically reduces rapidly (∼2 s) and settles within ∼10 min.

### Signal validation

To demonstrate that the CPEs were sensitive to systemic changes in O_2_ availability, the inspired O_2_ content was manipulated by applying gaseous O_2_ or nitrogen (N_2_) to the snout of freely-moving rats. The tissue O_2_ signal increased when O_2_ was applied and decreased when N_2_ was applied, and in both cases returned to baseline when the gas was removed (see Supporting Information, Fig. S1).

### Electrode calibration

At the end of the experiment, all CPEs were calibrated *ex vivo* by placing them in a bath of phosphate-buffered saline saturated with N_2_, then air and then O_2_, yielding a three-point calibration at known O_2_ concentrations of 0 μm, 240 μm and 1200 μm, respectively. The slope of the line through these three data points (using least-squares linear regression) yielded a calibration constant for each CPE, expressed in nanoamperes per micromolar (nA/μm). The mean calibration values were almost identical for the dHPC and vHPC electrodes (dHPC: 0.95 ± 0.05 nA/μm; vHPC: 0.94 ± 0.08 nA/μm). The measured amperometric currents were multiplied by these constants to obtain tissue O_2_ signals. Calibration allows for quantitative measurement of changes in tissue O_2_ concentration. For example, *in vivo*, a 5 nA signal increase in an electrode calibrated at 0.95 nA/μm would constitute a 4.75 μm increase in tissue O_2_. However, for a variety of reasons (e.g. Kane & O’ Neill, 1998), electrode responses *in vivo* may not be the same *ex vivo*, and in the present study O_2_ signals are described in arbitrary rather than quantitative units.

### Behavioural experiments

#### Experiment 1 – unconditioned anxiety on the black/white alley and food neophobia tests

Following the theoretical model of Gray, anxiety is defined as a state of goal conflict or uncertainty (Gray, 1982; Gray & McNaughton, 2000). Anxiety was investigated using two situational conflict tests: the black/white alley and food neophobia. In the black/white alley, the rat is initially placed into the black section, and latency to cross into the white section and time spent in each section can be used to index anxiety (i.e. more anxious rats are slower to enter and spend less time in the white section). In food neophobia, the rat is placed into a novel environment, and the time taken to commence eating a novel foodstuff is used to index anxiety (i.e. more anxious rats take longer to begin eating). Benzodiazepines reduce latency to enter the white section of the black/white alley (McHugh *et al.*, 2004) and latency to eat in the neophobia test (Shephard & Estall, 1984).

##### Apparatus

The black/white alley is based on the principles of the light/dark box (Crawley & Goodwin, 1980), and comprised a narrow, high-walled alley (1200 mm long, 100 mm wide, 300 mm high), divided into equal-sized black and white sections (each 600 mm, 100 mm wide, 300 mm high).

Food neophobia took place on a low-walled wooden T-maze (start arm: 800 mm long, 100 mm wide; goal arms: 600 mm long, 100 mm wide, elevated 450 mm from the floor). The rats were confined to a small section of one goal arm (240 mm long and 100 mm wide with a 30-mm-high surrounding wall) using a wooden block (300 mm high and 250 mm wide). A food well (15 mm high, 25 mm diameter; containing ∼15 reward pellets, 45 mg Rodent Diet Formula A/I; Noyes, Lancaster, NH, USA) was placed 40 mm from the exposed end of the arm.

##### Procedure

Rats were tested on the black/white alley approximately 2 weeks after surgery (*n*=8), and then food neophobia (*n*=7) 3 days later. The procedure was virtually identical for both tests. At the start of the session, the rat was placed in a holding cage (a cylindrical bowl 35 cm high, 56 cm open end diameter, 44 cm base diameter) and connected to the recording equipment. The potential was then applied and the rat left undisturbed for ∼30 min to allow the tissue O_2_ signal and arousal levels to settle. For the black/white alley test, the rat was then removed from the holding cage and placed into the black section of the black/white alley, facing the rear wall. Latency to cross into the white section, total time spent in each section, and crossings between each section were recorded. For the food neophobia test, the rat was removed from the holding cage and placed onto the T-maze, facing the block. Latency to begin eating was recorded. Each test lasted 600 s. Note that the food neophobia test was not terminated when the rat began eating (cf. McHugh *et al.*, 2004). Behaviour was recorded with a webcam synchronized to the tissue O_2_ signal. For the black/white alley test, the rats’ speed of movement and location were analysed off-line using custom-written software (Microsoft Visual Basic.NET; Microsoft, WA, USA). Speed and location were not calculated for the food neophobia test because the testing area was only just larger than the body length of the rat.

##### Data analyses

Tissue O_2_ signals in dHPC and vHPC during the anxiety tests were analysed with reference to a 60-s baseline recorded 30–90 s before testing began. During baseline recording, the rat was left undisturbed in the holding cage. We did not use the 30 s immediately before the test because the rat was removed from the holding cage during this period.

**Fig. 1:**
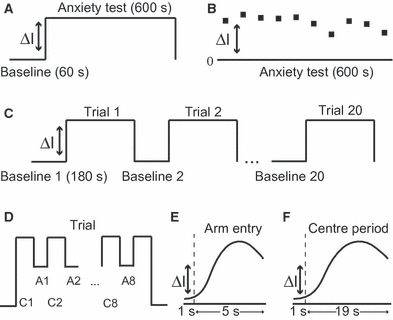
Summary schematics of analyses used for the anxiety tests (A and B) and radial maze working memory tasks (C–E). (A) The box-car approach used for the anxiety tests compared mean O_2_ signals in dHPC and vHPC during the test (600 s) with their respective 60-s baselines measured before the test. The difference between baseline and test is denoted by Δ*I*. (B) The second approach used for the black/white alley divided the 600-s test into 10 60-s time-bins, and used the O_2_ signal difference (Δ*I*=test signal − baseline signal) as the dependent variable. (C) The box-car approach used for the one-arm and eight-arm radial maze tasks compared mean dHPC and vHPC O_2_ signals from each trial with their respective baselines recorded before each trial. (D–F) The event-related analyses used to investigate the time course of signal change in dHPC. (D) Each trial was divided into the eight arm entries (A1, A2, …, A8) and eight periods in the centre of the maze (C1, C2, …, C8). The time course of O_2_ signal change in response to (E) entering an arm or (F) entering the central area was calculated relative to a 1-s baseline (left of the dotted line) measured immediately before centre–arm or arm–centre transitions.

The first analysis used a box-car approach to compare mean dHPC and vHPC tissue O_2_ signals during baseline and test (Fig. 1A). To this end, a repeated-measures anova was used with electrode placement (two levels: dHPC, vHPC) and task state (two levels: baseline, task) as within-subject factors. The primary purpose of the box-car approach was to determine if dHPC and vHPC were significantly more active during test than baseline. Box-car analyses were carried out separately for the black/white alley and food neophobia tests.

Next, we investigated correlations between the behavioural measures of anxiety and tissue O_2_ signal change from baseline (Δ*I* = test − baseline) in dHPC and vHPC. For the black/white alley, correlation coefficients were calculated for the following: (i) latency to enter white section vs. dHPC O_2_ signal; (ii) latency to enter white section vs. vHPC O_2_ signal; (iii) time spent in white vs. dHPC signal; (iv) time spent in white section vs. vHPC signal; (v) number of crossings between sections vs. dHPC signal; (vi) number of crossings vs. vHPC signal. As only one rat ate in the food neophobia test, no correlation coefficients were calculated.

In addition, we investigated tissue O_2_ signals when rats were in either the black or the (potentially more dangerous) white section of the black/white alley. This analysis used the tissue O_2_ signal change from baseline (Δ*I* = test − baseline) as the dependent variable in a repeated-measures anova, with electrode placement (two levels: dHPC, vHPC) and section (two levels: black, white) as within-subjects factors.

We also analysed the data on a finer temporal scale by dividing the 600-s trial into 10 60-s time-bins, and used the signal change from baseline (Δ*I* = test − baseline) as the dependent variable (Fig. 1B). In the black/white alley test we found that, across time-bins, the dHPC (but not vHPC) signal was strongly correlated with running speed. In other words, the dHPC tissue O_2_ signal was higher during time-bins when the speed of movement was high. We therefore wanted to use running speed as a covariate in this analysis. However, standard general linear approaches (e.g. ancova) are unable to deal with covariates that change over time (within-subjects covariates), and therefore we used a mixed linear model to analyse these data. Mixed models are increasingly used for repeated-measures analyses in biology, agriculture and medicine (e.g. Laird & Ware, 1982; Piepho *et al.*, 2003; Gueorguieva & Krystal, 2004; Howell, 2010). This analysis used electrode placement (two levels: dHPC, vHPC) and time-bin (10 levels) as repeated fixed factors, subject as a random factor, and speed as a time-varying covariate (10 speed measurements per rat, one for each level of time-bin) using a restricted maximum likelihood approach.

The food neophobia data were analysed with repeated-measures anova with electrode placement (two levels: dHPC, vHPC) and time-bin (10 levels) as within-subjects factors. Speed of movement was not measured in this test and therefore was not used as a covariate in this analysis.

Finally, in addition to the separate analyses of the black/white alley and food neophobia tests, we performed a combined analysis of both tests. This anova used within-subject factors of test (two levels: black/white alley, food neophobia), electrode placement (two levels: dHPC, vHPC) and time-bin (10 levels). Speed of movement was not used as a covariate in this combined analysis. The combined analysis had the advantage of increased statistical power, and was justified because both tests relied on situational conflict to generate anxiety and were virtually identical in procedure.

#### Experiment 2 – spatial working memory on the radial arm maze

##### Apparatus

The eight-arm radial maze was constructed from transparent acrylic (3 mm thick) with an octagonal central area (200 mm diameter) and eight arms (270 mm long, 100 mm wide, 280 mm high) radiating from each side. Doors (95 mm wide, 280 mm high) controlled access into and out of the arms, and were operated by the experimenter using a pulley system that ran underneath each door, such that the doors dropped down beneath the maze when opened. Steel food wells (12 mm diameter, 10 mm high) were placed 20 mm from the end of each arm. The maze was elevated 700 mm from the floor in a room containing various prominent extra-maze cues, including a bench, electrical equipment and wall posters.

##### Procedure

Nine rats took part in Experiment 2, which began approximately 3 weeks after surgery. Eight of the nine rats had previously taken part in Experiment 1. Four rats were trained on the eight-arm spatial working memory task, and five were trained on the one-arm control task, including the one rat with bilateral dHPC CPEs. At the start of each session, the rat was placed in a holding cage in the testing room and connected to the recording equipment. The potential was then applied and the rat left undisturbed for ∼30 min. The rat was then removed from the holding cage and placed on the central area of the radial maze. An opaque plastic bucket (29 cm high, 25 cm open end diameter, 20 cm base diameter) was then placed over the rat for 180 s. This period served as the baseline for the first trial, and the removal of the bucket signalled the start of the trial.

##### Eight-arm task

In the eight-arm task, all eight arms were baited at the start of each trial, and the rats were required to adopt a win-shift strategy to collect all eight rewards (one sucrose pellet, Rodent Diet Formula P; Noyes). Between each arm choice the doors were held in the closed position for 20 s to increase the spatial working memory demand and prevent rats from making successive entries to adjacent arms. Re-entering an arm previously visited on the same trial constituted a working memory error and was not rewarded. Reference memory errors (i.e. entering never-baited arms) could not be made because every arm was baited at the start of each trial. A flow diagram of the eight-arm task is given in Supporting Information (Supporting Information, Fig. S2).

##### One-arm task

In the one-arm task, one arm (e.g. ‘west’) was designated the reward arm and the remaining seven arms were always in the closed position. The reward arm remained constant throughout training. Rats in the one-arm group were required to enter the reward arm, consume the reward, and then return to the central area of the maze; and to do this eight times per trial. The door of the reward arm was held in the closed position for 20 s between each arm entry. No working memory errors could be made because the same arm was always rewarded, and no reference memory errors could be made because the other seven maze arms were always blocked.

Upon completion of a trial in either the eight-arm or one-arm tasks (i.e. when the rat returned to the central area after collecting the final reward), the bucket was again placed over the rat. This signalled the end of the trial and the start of the 180-s inter-trial interval (ITI). Between four and 10 trials were conducted in each session and the rat remained on the maze for the whole of a session, either under the bucket or performing the task. Every trial was recorded with a webcam that was synchronized to the tissue O_2_ signal, and the rats’ speed and location were analysed off-line using custom-written software. All rats performed at least 30 trials.

##### Data analyses

Initially, analyses of tissue O_2_ signals were carried out on data collected during the last 20 training trials when rats in the eight-arm group were at a good level of working memory performance (mean: 1.0 ± 0.3 error per trial). These data were collected over four to five sessions. By this time, both groups of rats were thoroughly habituated to the maze and showed no signs of anxiety. These data were analysed in two ways: (i) box-car; and (ii) event-related.

The box-car design was used to compare mean dHPC and vHPC signals during baseline and trial periods. The mean signal during the ITI (when the rat was under the bucket) served as the baseline period for the subsequent trial (Fig. 1C). An anova investigated the effects of task state (two levels: task, baseline), memory load (two levels: eight-arm, one-arm task) and electrode placement (two levels: dHPC, vHPC). Eight rats with dHPC/vHPC electrodes were used in this analysis.

Next, we used an event-related approach by dividing each trial into periods of time spent in the central area and time spent in the arms of the maze (Fig. 1D). In these analyses, we calculated signal changes over time separately for: (i) the first eight ‘arm entries’; and (ii) the corresponding eight ‘centre periods’ before (and between) each arm entry. Each time series was calculated relative to a local baseline measured 1 s immediately before an arm or centre transition. Note that the first period in the centre was not strictly an arm–centre transition because it occurred at the start of each trial when the bucket was removed from over the rat. Rats were free to spend as much time as they wanted in the arms, but spent at least 5 s, so arm entry time series were calculated over 5 s from the point at which the rat entered an arm (Fig. 1E). Note that in most cases this 5-s period would include the time taken to enter the arm, reach the foodwell and consume the reward, but not the return journey to the centre. Rats were confined to the central area for 20 s between arm entries, but we calculated centre period time series over 19 s to ensure that arm entry periods were not included (Fig. 1F). The temporal resolution for these event-related changes was 1 data point/s. Only signals from the dHPC were used for the event-related analyses and nine rats were included (only the left dHPC electrode was used from the bilaterally implanted dHPC rat). For arm entries, an anova was performed with memory load (two levels: eight-arm, one-arm) as a between-subjects factor, and arm entry (eight levels: entries 1–8) as a within-subjects factor. For centre periods, an anova was performed with memory load as a between-subjects factor, and centre period (eight levels: periods 1–8) and time-bin (19 levels: 1st–19th second) as within-subjects factors.

Having determined a difference between dHPC O_2_ signals in the eight-arm and one-arm tasks during the last 20 trials, we then investigated if this difference was present early in training. In addition, in the previous analysis, to achieve a good level of performance rats in the eight-arm group had completed more trials than those in the one-arm group. Therefore, we also investigated if the difference was present at a later stage of training when the eight-arm and one-arm groups were matched for the number of trials completed. To this end, we compared the eight-arm and one-arm groups early (trials 1–5) and late in training (trials 26–30). Blocks of five trials were used because of signal-to-noise issues when analysing single trials. Again, we calculated arm entry time series over 5-s epochs from the point at which the rat entered a given arm, using a temporal resolution of 1 data point/s (Fig. 1E). These data were then analysed in an anova with memory load (two levels: eight-arm, one-arm) as a between-subjects factor, and training stage (two levels: trials 1–5, trials 26–30), entry (eight levels: entry 1–8) and time-bin (five levels: 1st–5th second) as within-subjects factors.

#### Experiment 3 – tissue O_2_ and LFPs during sleep and waking

To investigate the extent to which dHPC O_2_ signals were related to physical activity, we recorded locomotor activity, dHPC tissue O_2_ and LFPs simultaneously in a separate group of rats (*n*=4) during sleep and waking.

##### Apparatus

All recordings took place in the holding cage in the testing room.

##### LFP recordings

Intra-HPC LFPs were recorded using a differential amplifier in dc mode (DP-301, Warner Instruments, CT, USA), filtered (high-pass = 0.1 Hz; low-pass = 1 kHz), amplified and sampled continuously at 1 kHz. At the start of each recording session the amplifier was calibrated using a 1-mV square wave as per the manufacturer's instructions.

##### Procedure

At the start of each session, rats were placed in the holding cage and connected to the recording equipment. The potential was then applied and data collection began 30 min later. LFPs, tissue O_2_ and locomotor activity levels were then monitored continuously for 4 h.

##### Data analyses

Assessment of sleep or waking state was based on a validated computer-based sleep-scoring algorithm that used locomotor activity and filtered LFP amplitudes to assign the rat to one of four behavioural states: active wake; quiet wake; rapid eye movement (REM) sleep; or non-REM (NREM) sleep (Louis *et al.*, 2004). Locomotor activity levels were recorded with a webcam, and assessed using custom-written software (MATLAB vR2007a, MathWorks, MA, USA) that detected thresholded pixel differences for pairs of consecutive frames and then calculated the summed activity scores for each 5-s epoch over the 4-h recording duration (2880 epochs per rat). LFPs were digitally filtered (Chart®, AD Instruments) into the following frequency bands: delta (1.5–6 Hz), theta (6–10 Hz), alpha (10.5–15 Hz), beta (22–30 Hz) and gamma (35–45 Hz). These bands were rectified and the average amplitude calculated for each 5-s epoch.

The sleep-scoring algorithm had three stages. In the first stage, if the locomotor activity level was over threshold then an active wake state was assigned. If locomotor activity was below threshold (i.e. if the rat was not moving) then the algorithm calculated the amplitude ratio of (delta × alpha)/(beta × gamma); if this was over threshold then a state of NREM sleep was assigned. If this ratio was below threshold then a further test of the amplitude ratio for (theta^2^/delta × alpha) was performed; if over threshold then a REM sleep state was assigned, if under threshold then a quiet wake state was assigned. These data were then visually inspected for sections of REM that did not follow NREM sleep or those with duration < 25 s (i.e. fewer than five consecutive REM epochs). Where REM periods were flanked by active wake epochs or by active wake before and NREM after (or vice versa), the REM assignment was changed to ‘quiet wake’. Isolated REM epochs flanked by NREM periods were re-assigned to NREM. Even before manual reassignment, this algorithm has high concordance (78–88%) with experienced human sleep scorers (Louis *et al.*, 2004).

dHPC O_2_ signals were processed as follows. First, to eliminate signal drift over the 4-h recording period, the linear trend (least-squares regression method) was subtracted from the O_2_ signal (Challis & Kitney, 1990); then the average dHPC tissue O_2_ signal was calculated for each 5-s epoch. Next, average O_2_ signals were calculated for each of the four states: active wake, quiet wake, NREM and REM. Finally, to enable comparison across different animals, the mean of the de-trended data was subtracted from the tissue O_2_ value for each behavioural state. This yielded a tissue O_2_ difference score for each of the four states. These data were analysed using a one-way anova, with behavioural state (four levels: active wake, quiet wake, NREM, REM) as a within-subjects factor.

### Statistical procedures

Data were analysed using anova or a mixed linear model in spss (version 15, spss, IL, USA). anovas are described using a modified version of Keppel's (1982) notation in which the dependent variable is defined in the form: *A*_2_ × *B*_3_ × *S*_8_, where *A* is a factor with two levels, *B* a factor with three levels, and *S*_8_ denotes there were eight subjects in the analysis. Interactions were investigated using simple main effects, and all tests were performed with the familywise error rate at *α*= 0.05. All graphs show the mean ± 1 standard error of the mean (SEM).

### Histology

After the final session of radial maze testing, rats were injected with Euthatal (200 mg/mL sodium pentobarbitone; 200 mg/kg) and perfused transcardially with physiological saline (0.9% NaCl) followed by 10% formol saline (10% formalin in 0.9% NaCl). Their brains were removed and placed in 10% formol saline for 3 days, and then transferred to a 30% sucrose–formalin solution for 24 h and frozen. Coronal sections (50 μm) were then cut on a freezing microtome and stained with Cresyl violet to enable visualization of the electrode tracts.

## Results

### Histology

**Fig. 2:**
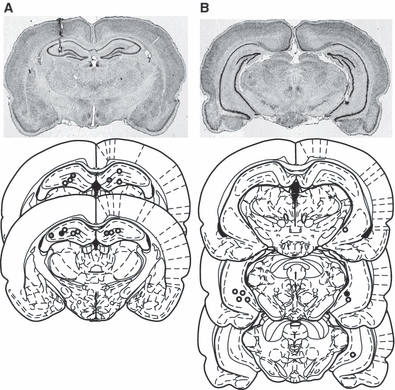
Photomicrographs and reconstructions of electrode placements in (A) dHPC and (B) vHPC. Coronal sections for the reconstructions are taken from the atlas of Paxinos & Watson (1998), plates 33–34 (dHPC) and plates 38, 42, 44 (vHPC). The rat shown in the photomicrograph is indicated by the dark-filled circles in the reconstructions. Tissue O_2_ electrodes are shown with open circles, and LFP (and dual tissue O_2_/LFP) electrode positions are shown with grey-filled circles.

dHPC and vHPC electrodes were predominantly located around the CA3 field. Reconstructions and a representative photomicrograph from one rat are shown in Fig. 2. There were no systematic differences in the tissue O_2_ signals based on differences in subfield placement within a subregion, and no rats were excluded on histological grounds.

### Experiment 1 – anxiety on the black/white alley and food neophobia tests

#### Behavioural data

**Fig. 3:**
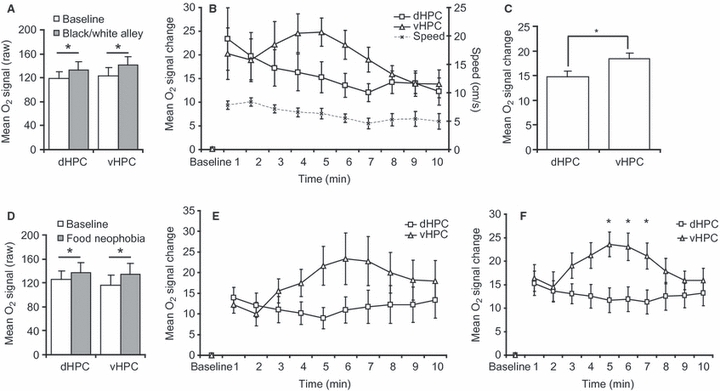
Tissue O_2_ signals during the black/white alley (A–C) and food neophobia anxiety tests (D and E), and combined (F). (A) Black/white alley box-car analysis – mean (± SEM) baseline and test O_2_ signals for dorsal hippocampus (dHPC) and ventral hippocampus (vHPC). The overlap in the error bars between baseline and the test signals reflects between-subjects variance in the raw signals and not within-subjects variance – all rats had higher dHPC and vHPC signals during the test than baseline. (B) Black/white alley time-binned analysis – mean O_2_ (± SEM) signal change from baseline (Δ*I*) in dHPC and vHPC divided into 60-s time-bins. Mean (± SEM) running speed is plotted below the O_2_ signals and against the right hand *y*-axis. (C) Black/white alley – mean (± SEM) O_2_ signal change from baseline in the dHPC and vHPC after controlling for running speed. (D) Food neophobia box-car analysis – mean (± SEM) baseline and test O_2_ signals for dHPC and vHPC. (E) Food neophobia time-binned analysis – mean O_2_ (± SEM) signal change from baseline (Δ*I*) in dHPC and vHPC divided into 60-s time-bins. (F) Combined analysis for black/white alley and food neophobia tests – mean O_2_ (± SEM) signal change from baseline (Δ*I*) in dHPC and vHPC. *P<0.05.

In both the black/white alley and food neophobia tests, the rats displayed behaviour consistent with anxiety. In the black/white alley, they were slow to cross into the white section (mean latency = 159 ± 78 s; mean number of crossings = 10 ± 3), showed a strong preference for the black over the white section (black = 87%, white = 13%), and engaged in risk assessment behaviours, such as rearing and stretch-attend postures (Rodgers *et al.*, 1999). They were more active at the start than the end of the test, as indicated by a decrease in mean running speed across the trial (Fig. 3B, dotted line). In the food neophobia test, only one rat ate any of the food pellets, precluding any quantitative analysis.

#### Amperometric O_2_ responses during anxiety – box-car analyses

In both anxiety tests, tissue O_2_ signals in both dHPC and vHPC were higher during the task than the baseline period (Fig. 3A and D; note that the overlap in the error bars between the baseline and the task signals reflects between-subjects variance in the raw signals and not within-subjects variance). Analysis of the black/white alley (anova– electrode placement_2_ × task state_2_ × *S*_8_) revealed a significant effect of task state (*F*_1,7_ = 126.9, *P*=0.001), reflecting higher signals during the task than during baseline, but no effect of electrode placement (*F*_1,7_=0.3, *P*=0.6) or interaction (*F*_1,7_=0.6, *P*=0.5). Similarly, analysis of the food neophobia test (anova– electrode placement_2_ × task state_2_ × *S*_7_) revealed a significant effect of task state (*F*_1,6_=38.1, *P*=0.001), but no effect of electrode placement (*F*_1,6_=0.1, *P*=0.8) or interaction (*F*_1,6_=1.7, *P*=0.2). In summary, when mean signals over the whole anxiety task were considered, both dHPC and vHPC were activated significantly above baseline and to an equivalent extent.

#### Amperometric O_2_ responses during anxiety – correlations with behaviour

Next, we calculated correlation coefficients between the behavioural indices of anxiety in the black/white alley test (latency to cross into the white section, time spent in the white section, crossings between sections) and tissue O_2_ signal change from baseline (test − baseline) in dHPC and vHPC. There were no significant correlations between behavioural indices and tissue O_2_ signals in either dHPC or vHPC (all *r*^2^ < 0.2, *P*>0.2). Only one rat ate during food neophobia, so no correlations were calculated for this test.

We also investigated dHPC and vHPC tissue O_2_ signals when rats were in either the black section or the white section of the black/white alley (anova– electrode placement_2_ × section_2_ × *S*_7_). We found no effect of electrode placement (*F*_1,6_=0.6, *P*=0.46), but there was an effect of section (*F*_1,6_=7.6, *P*=0.03), with higher signals when rats were in the white section. There was no interaction (*F*_1,6_=0.4, *P*=0.54), but it is noteworthy that a significant difference between the white and black sections was only present in the vHPC tissue O_2_ signal (*F*_1,6_=8.3, *P*=0.03) and not the dHPC (*F*_1,6_=1.5, *P*=0.3).

#### Amperometric O_2_ responses during anxiety – time-bin analyses

Although there were no differences between the mean dHPC and vHPC tissue O_2_ signals over the whole test period, there was a clear difference in the time course of the dHPC and vHPC signals when we divided each test into 10 60-s time-bins (Fig. 3B and E). Moreover, with the data in this form it was apparent that running speed in the black/white alley was strongly correlated with the dHPC O_2_ signal (*r*^2^=0.84; *t*_8_=6.5, *P*=0.001), but not the vHPC O_2_ signal (*r*^2^=0.17; *t*_8_=1.3, *P*=0.2). Therefore, to control for the influence of running speed, the black/white alley data were re-analysed with a mixed linear model with electrode placement (dHPC, vHPC) and time-bin (10 levels) as fixed factors, and running speed as a time-varying covariate (10 levels). This revealed a significant effect of electrode placement (*F*_1,7_=5.6, *P*=0.05), with higher signals in vHPC than dHPC (Fig. 3C). There was no effect of time-bin (*F*_9,63_=1.4, *P*=0.2) or interaction (*F*_9,63_=0.7, *P*=0.7).

Analysis of the time-binned food neophobia tissue O_2_ data (anova– electrode placement_2_ × timebin_10_ × *S*_7_) revealed a significant interaction between electrode placement and time-bin (*F*_9,54_=3.2, *P*=0.004), but no main effects of electrode placement (*F*_1,6_=1.3, *P*=0.3) or time-bin (*F*_9,54_=1.2, *P*=0.3). The interaction appeared to be driven by higher vHPC than dHPC tissue O_2_ signals during time-bins 4–7 (Fig. 3E), but this was statistically underpowered, and simple main effects analysis did not identify specific time points where vHPC signals were significantly higher than dHPC (largest difference was the 5th timebin –*F*_1,6_=4.2, *P*=0.09).

Therefore, to increase statistical power, we performed a combined analysis of tissue O_2_ signals from the black/white alley and food neophobia tests (anova– test_2_ × electrode placement_2_ × timebin_10_× *S*_7_). This revealed a significant electrode placement × time-bin interaction (*F*_9,54_=3.1, *P*=0.005), and analysis of simple main effects revealed higher vHPC than dHPC O_2_ signals at time points 5 (*F*_1,6_=7.0, *P*=0.04), 6 (*F*_1,6_=6.8, *P*=0.04) and 7 (*F*_1,6_=6.6, *P*=0.04). These data are shown in Fig. 3F.

In summary, compared with the pre-test baseline, the black/white alley and food neophobia anxiety tests elicited a significant (and equivalent) tissue O_2_ signal increase in both dHPC and vHPC. However, in both anxiety tests there were markedly different time courses for the dHPC and vHPC O_2_ signals. Moreover, although neither signal was correlated with the behavioural measures of anxiety in the black/white alley test, only the dHPC signal was correlated with running speed, which suggests different influences on the dHPC and vHPC signals. When running speed was used as a covariate, there were significantly higher signals in vHPC than dHPC. Moreover, analysis of the food neophobia test and the combined analysis found significant interactions between electrode placement and time-bin, with higher signals in vHPC. Collectively, these data support a preferential role for vHPC in anxiety.

### Experiment 2 – spatial working memory on the radial maze

#### Behavioural data

Rats in the eight-arm group made fewer working memory errors as training proceeded. Over the last 20 trials of training they averaged approximately one working memory error per trial (mean – 1.01 ± 0.32). No errors could be made in the one-arm task, precluding an objective measure of performance, but all rats in this group learned the task requirements within the first couple of trials (i.e. entering the arm, running to the foodwell, collecting the reward and returning to the central area).

#### Amperometric O_2_ responses during spatial processing – box-car analyses

**Fig. 4:**
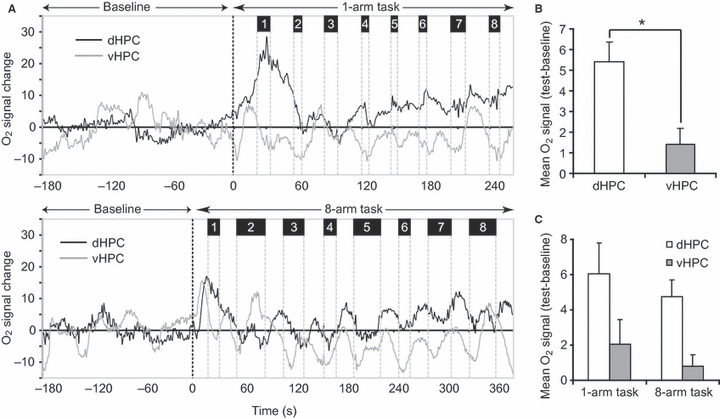
Tissue O_2_ signals in dHPC and vHPC during the radial maze tasks. (A) Raw data from representative rats in the one-arm (upper panel) and eight-arm tasks (lower panel) during baseline (left of black vertical dotted line) and task performance (right of black vertical dotted line). Grey vertical dotted lines show transitions into and out of the maze arms. The numbers at the top of the figure show arm entries 1–8 in each trial. dHPC traces are in black, vHPC traces are in gray. (B) Mean (± SEM) O_2_ signal change from baseline in dHPC and vHPC combined across the eight-arm and one-arm tasks. (C) Mean (± SEM) O_2_ signal change from baseline split by subregion and task. *P=0.001.

Figure 4A shows raw tissue O_2_ data examples from the one-arm (upper panel) and eight-arm (lower panel) tasks, and illustrates three general observations. First, in both the one-arm and eight-arm tasks, dHPC tissue O_2_ signals were consistently higher during task performance than baseline. In contrast, vHPC signals were not consistently higher than their baseline in either task. Second, there were no obvious differences between the responses evoked by the eight-arm and one-arm tasks when comparing mean signal levels over whole trials. Third, in both tasks there was a large increase in the dHPC signal at the start of each trial (i.e. following removal of the bucket).

Data from the eight-arm and one-arm tasks were first analysed using a box-car approach comparing mean dHPC and vHPC O_2_ signals in each trial to their respective baseline periods [anova– memory load (eight-arm vs. one-arm)_2_ × task state (baseline vs. trial)_2_ × electrode placement_2_ × *S*_8_)]. There was a main effect of task state (*F*_1,6_=18.8, *P*=0.005), with tissue O_2_ signals significantly higher during trials than the baseline, and a task state × electrode placement interaction (*F*_1,6_=26.5, *P*=0.002). Simple main effects analysis of this interaction revealed that the effect of task state was only present in the dHPC signal (*F*_1,6_=30.9, *P*=0.001) and not the vHPC signal (*F*_1,6_=3.4, *P*=0.1). A direct comparison of task-related dHPC and vHPC signals was carried out by calculating the mean signal change from baseline (Δ*I*) for each brain region and comparing these in a paired samples *t*-test. This revealed a significantly greater signal increase in dHPC than vHPC (*t*_7_=5.6, *P*=0.001; Fig. 4B). However, using the box-car approach, there was no main effect of memory load or any significant interactions involving memory load (all *F*<1; NS; Fig. 4C). In summary, the box-car approach found a greater signal increase in dHPC than vHPC, but equivalent signals in the eight-arm and one-arm tasks.

#### Event-related tissue O_2_ analyses of the eight-arm and one-arm tasks

By using the mean signal level over the whole trial as the dependent variable, the box-car analysis ignored the dynamics of the O_2_ signal within each trial. Therefore, the next analysis used an event-related approach to investigate the dHPC O_2_ signal on a finer temporal scale to see if differential signals were evoked by the eight-arm and one-arm tasks at any point during the trial. We divided each trial into epochs based on the eight-arm entries and the eight periods in the central area, and plotted the time course of signal change for arm–centre or centre–arm transitions. The baseline for these signal changes was the 1 s before each transition. We restricted these analyses to the dHPC because the previous analysis found no significant increase of vHPC above baseline during the radial maze tasks.

**Fig. 5:**
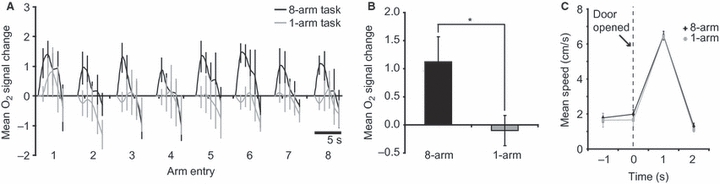
Event-related tissue O_2_ signal changes in dHPC during arm entries in the eight-arm (black lines/bars) and one-arm (grey lines/bars) tasks. (A) Time course of dHPC O_2_ signals (mean ± SEM) during the first 5 s of arm entry for the first eight entries of a trial. Signal changes were calculated relative to a 1-s baseline measured immediately before centre–arm transitions. (B) Mean (± SEM) O_2_ signals in dHPC for the eight-arm and one-arm tasks combined across the eight entries. (C) Mean (± SEM) running speed for the 2 s before and 2 s after arm entries in the one-arm and eight-arm tasks combined across the eight entries. *P=0.04.

With the data in this event-related form, there was a clear difference between the eight-arm and one-arm tasks when rats entered the arms of the maze. This was characterized by a transient but consistent increase in the dHPC O_2_ signal in the eight-arm but not the one-arm task (Fig. 5A). To analyse these data we used the mean dHPC O_2_ signal during the first 1 s of arm entry for each of the eight-arm entries in a trial (anova– memory load_2_ × arm entry_8_ × *S*_9_). This analysis revealed a main effect of memory load (*F*_1,7_=6.2, *P*=0.04), with significantly higher dHPC signals in the eight-arm than one-arm task (Fig. 5B), but no effect of arm entry number (*F*_7,49_=1.1, *P*=0.37) or interaction (*F*_7,49_=1.7, *P*=0.13). Importantly, the dHPC O_2_ signal difference between the eight-arm and one-arm tasks could not be explained by differences in running speed when rats entered the arms of the maze, because this was almost identical in the two groups (Fig. 5C). An analysis of running speeds for the 2 s before and 2 s after arm entries (anova– memory load_2_ × time-bin_4_ × *S*_9_) found no effect of task/memory load (*F*_1,7_=0.4, *P*=0.5) or interaction (*F*_3,21_=0.1, *P*=0.96), although there was a main effect of time-bin (*F*_3,21_=232.9, *P*<0.001), which reflected the increase in speed when rats ran into the arms, but this was equivalent in the eight-arm and one-arm tasks.

**Fig. 6:**
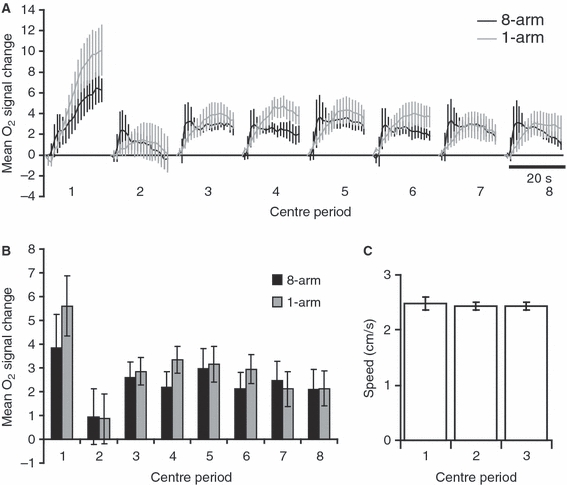
Event-related tissue O_2_ signal changes in dHPC during periods in the centre of the maze for the eight-arm (black lines/bars) and one-arm (grey lines/bars) tasks. (A) Time course of dHPC O_2_ signals (mean ± SEM) during the eight periods in the centre of the maze (e.g. centre period 1 is before the first arm entry). Signal change was calculated relative to a 1-s baseline measured immediately before arm–centre transitions (except for the first period when the baseline was the final 1 s of the ITI). (B) Mean (± SEM) dHPC O_2_ signals for each centre period (i.e. average signal over the 19 s). (C) Mean (± SEM) running speed for the first three periods in the centre.

In contrast to arm entries, there were no clear dHPC O_2_ signal differences between the eight-arm and one-arm groups during the ‘centre periods’, although signals were slightly higher in the one-arm group (Fig. 6B). However, in both groups, dHPC O_2_ signals were higher during the first period in the centre compared with periods 2–8 (Fig. 6A and B). Analysis of these data (anova– memory load_2_ × centre period number_8_ × time-bin_19_ × *S*_9_) revealed no effect of memory load (*F*_1,7_=0.2, *P*=0.68), but there were effects of centre period number (*F*_7,49_=11.7; *P*=0.001) and time-bin (*F*_18,126_=6.1, *P*=0.0001), and a centre period number × time-bin interaction (*F*_126,882_=11.6, *P*=0.001). The effect of centre period number reflected a higher dHPC signal for the first period in the centre compared with periods 2–8 (all *P*<0.05), and lower signals in the second period compared with the others (all *P*<0.05; except second vs. seventh: *P*=0.053). Again, we investigated if this dHPC O_2_ signal difference could be explained by differences in running speeds (Fig. 6C), but limited this analysis to centre periods 1–3 because these periods were different from one another in the tissue O_2_ analysis. This analysis (anova– memory load_2_ × centre period_3_ × *S*_9_) found no effect of task (*F*_1,7_=1.9, *P*=0.2), centre period number (*F*_2,14_=0.1, *P*=0.9) or interaction (*F*_2,14_=0.1, *P*=0.95), confirming that running speed could not explain differences in the dHPC tissue O_2_ signal.

**Fig. 7:**
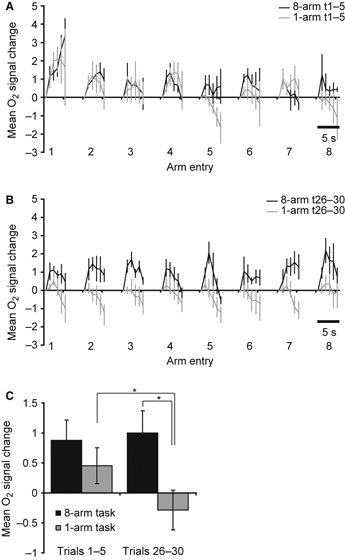
Event-related tissue O_2_ signal changes in dHPC during arm entries early and late in training. (A) Early training (*t*_1–5_): time course of dHPC O_2_ signals (mean ± SEM) during the first 5 s of arm entry for the first eight entries of a trial for the eight-arm (black) and one-arm (grey) tasks. (B) As in (A), but for late training trials (*t*_25–30_). (C) Mean (± SEM) dHPC O_2_ signals showing the interaction between memory load and training stage. *P<0.05.

The event-related analyses above established that dHPC tissue O_2_ signals were higher in the eight-arm than the one-arm task when rats entered the arms of the maze. To see if this difference was present: (i) at the start of training; and (ii) when the eight-arm and one-arm groups were matched for the number of trials completed, we compared responses early (trials 1–5) and late in training (trials 26–30). Error rates in the eight-arm group were 4.5 (± 1.0) errors per trial for early training and 2.0 (± 0.3) errors per trial for late training. As before, we calculated the dHPC tissue O_2_ signal (for 5 s from the point of entry) for the eight-arm entries. Early in training, there were no differences between the eight-arm and one-arm groups, but later in training dHPC tissue O_2_ signals were lower in the one-arm group (Fig. 7A and B). Analysis of these data (anova– memory-load_2_ × training stage_2_ × entry_8_ × time-bin_5_ × *S*_9_) revealed a significant memory load × training stage interaction (*F*_1,7_=8.6, *P*=0.02). Simple main effects analysis found that this interaction was driven by an effect of training stage in the one-arm group (*F*_1,7_=14.3, *P*=0.01; with higher signals early in training), but not the eight-arm group (*F*_1,7_=0.3, *P*=0.6); and also an effect of memory load in the later training trials (*F*_1,7_=6.6, *P*=0.04; with higher signals in the eight-arm than the one-arm task) but not in the early trials (*F*_1,7_=0.9, *P*=0.4). This interaction is shown in Fig. 7C. Thus, the difference between the eight-arm and one-arm tasks during arm entries was not present initially but emerged over training, and was driven by reduced dHPC tissue O_2_ responses in the one-arm group during late training. In other words, repeatedly entering the same arm on each and every trial reduced the dHPC tissue O_2_ response. In contrast, there was no difference in the eight-arm group between the early and late training phases.

In summary, the box-car analysis of the radial maze tasks revealed higher tissue O_2_ signals in dHPC than vHPC. Second, although the box-car analysis did not find any differences between the eight-arm and one-arm tasks, the event-related analyses revealed a clear difference between the eight-arm and one-arm groups in the dHPC tissue O_2_ signal when rats entered the arms of the maze, an effect that could not be explained by differences in running speed. Moreover, when rats were in the centre of the maze there was no statistical difference between the eight-arm and one-arm groups, but signals in the one-arm group were numerically higher, which may explain why the box-car analysis found no difference between the two tasks overall. In addition, in both tasks the dHPC tissue O_2_ signal was significantly higher during the first centre period compared with the others; and this also could not be explained by differences in running speed. Finally, the difference between the eight-arm and one-arm tasks during arm entries was not present at the start of training but emerged later in training, and was driven by a reduction in dHPC tissue O_2_ signals in the one-arm group.

### Experiment 3 – tissue O_2_ and LFPs during sleep and waking

**Fig. 8:**
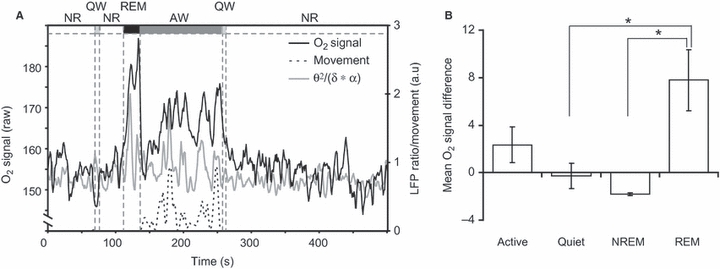
dHPC tissue O_2_ signals during sleep and waking. (A) Representative trace from one rat showing dHPC tissue O_2_, local field potential (LFP) ratio (theta2/delta × alpha), and movement data. AW, active waking; NR, non-rapid eye movement; QW, quiet waking. (B) Mean (± SEM) dHPC O_2_ signals for the four behavioural states: active waking, quiet waking, NREM and REM. *P<0.05.

In the previous experiments, dHPC tissue O_2_ signals were influenced by physical activity, although ultimately the differences between the eight-arm and one-arm conditions could not be explained by differences in physical activity. Nevertheless, over the course of our studies we also noticed large increases in dHPC tissue O_2_ signals when the rats were asleep in the holding cage, confirming that changes in dHPC tissue O_2_ were not dependent simply upon physical activity. To investigate this formally, we recorded tissue O_2_ and LFPs simultaneously from the dHPC during sleep and waking in a separate group of rats. During a continuous 4-h recording session, rats spent 18.5% (± 3.1) of their time in active waking, 7.4% (± 1.7) in quiet waking, 64% (± 2.6) in NREM sleep and 9.8% (± 1.1) in REM sleep. Figure 8A shows a representative example from one rat showing periods of quiet wake, active wake, NREM and REM sleep. Tissue O_2_ signals were typically highest during REM sleep and active waking periods. To analyse these data we calculated mean tissue O_2_ levels during each of the four states and expressed these as difference scores from the mean O_2_ signal recorded (and averaged) over the whole session. The highest tissue O_2_ levels were seen during REM sleep, then active wake, then quiet wake and then NREM sleep (Fig. 8B). One-way anova (state_4_ × *S*_4_) found a main effect of state (*F*_3,9_=6.2, *P*=0.02), and *post hoc* comparisons revealed significantly higher O_2_ signals during REM sleep compared with NREM and quiet waking (*P*<0.05), but not active waking (*P*=0.3). There were no other significant differences. In summary, dHPC tissue O_2_ signals were not dependent solely upon physical activity as the largest signals were seen during REM sleep.

## Discussion

### Summary of results

Our results demonstrate a double-dissociation of function in the HPC, with increased vHPC tissue O_2_ signals during anxiety and increased dHPC tissue O_2_ signals during spatial processing. These data are consistent with lesion studies in rodents and functional neuroimaging studies in humans, and illustrate the potential of CPA to bridge the gap between these very different methodologies. In addition, our data demonstrate that dHPC tissue O_2_ signals are modulated by spatial memory demands, with higher signals during the eight-arm radial maze task compared with the one-arm control task. This difference was restricted to the time point when rats entered the arms of the maze and was not present at the start of training, but rather it emerged later and was driven by a reduction in the dHPC tissue O_2_ signal in the one-arm group as training proceeded. This result is consistent with, and expands upon, previous findings showing higher IEG expression in rat HPC following the eight-arm vs. the one-arm task (Vann *et al.*, 2000).

Moreover, two important findings demonstrate that dHPC tissue O_2_ signals are not simply a reflection of physical activity. First, differences in running speed did not explain differences in the tissue O_2_ signal ‘between’ the eight-arm and one-arm groups during arm entries; nor did they explain dHPC O_2_ signal differences across arm entries ‘within’ each group for periods in the centre of the radial maze. Second, the largest dHPC O_2_ signals were seen during REM sleep in the absence of any physical activity.

### A preferential role for vHPC in anxiety

vHPC tissue O_2_ signals were significantly higher than baseline during the anxiety tasks, but were not higher than baseline during spatial processing on the radial maze. These findings are consistent with evidence from human functional imaging and rodent lesion studies. In humans, sustained anxiety evokes significant activation of anterior HPC (Hasler *et al.*, 2007), and in rats reduced anxiety is a consequence of vHPC (but not dHPC) lesions in a variety of tests, including those used in the present study (Bannerman *et al.*, 2002; Kjelstrup *et al.*, 2002; McHugh *et al.*, 2004; Pentkowski *et al.*, 2006). In contrast, vHPC lesions have little effect on tasks of spatial memory (Moser *et al.*, 1995; Bannerman *et al.*, 1999; Pothuizen *et al.*, 2004). This preferential role for the vHPC in anxiety is consistent with its strong reciprocal connectivity with the amygdala and other structures on the hypothalamic–pituitary–adrenal (HPA) axis that have long been identified with emotional processing (Krettek & Price, 1977; van Groen & Wyss, 1990; Canteras & Swanson, 1992; Risold & Swanson, 1996; Pikkarainen *et al.*, 1999).

However, in the present study, tissue O_2_ levels in vHPC did not correlate with behavioural indices of anxiety in the black/white alley test, which requires further discussion. There are several possible explanations for this finding. First, most rats exhibited an anxious phenotype on this task, and the lack of correlation could be due to a ceiling effect and/or the lack of a range of anxiety levels across the group of animals. Second, with only eight rats, these correlations were statistically underpowered and likely affected by behavioural scores at the extreme ends of the scale. For example, when inclusion criteria were restricted to rats with latencies lying between the 25th and 75th percentiles (*n*=5), there was a strong correlation between latency to enter the white section and tissue O_2_ signals in vHPC (*r*^2^=0.8; *t*_3_=4.6, *P*=0.02). Excluding data in this way is, of course, unjustifiable, but this example illustrates the effect of removing three data points on correlation coefficients in small samples. Third, the behavioural indices used in anxiety tasks can be influenced by factors independent of anxiety (e.g. general locomotor activity). This does not invalidate the task as an animal model of anxiety, but it does mean that the scores of individual rats must be interpreted with caution. Fourth, it is possible that tissue O_2_ signals in vHPC may not be able to discriminate state anxiety levels between individual subjects. In any event, our thesis is one of preferential involvement of vHPC over dHPC in a brain system (or systems) associated with anxiety, and not that the vHPC alone is the exclusive neural substrate of anxiety.

A further potential conundrum rests on the assumption that rats’ anxiety levels are likely to be highest at the start of the test and then gradually reduce over time; and that if vHPC tissue O_2_ signals serve as an index of anxiety, then they too should follow this pattern. But we did not see this pattern, and in both anxiety tests the vHPC signal had an inverted U-shaped response – increasing after the first few minutes, typically reaching a maximum after 5 or 6 min, and declining thereafter. However, it is simply not possible to know when anxiety levels are highest during these tests. As previously stated, in psychological terms anxiety is defined as the response to the conflict between competing goals or response options (e.g. approach/avoid), and is not a response to the specific danger or threat cues themselves (Gray & McNaughton, 2000). Both tests exploit this approach/avoidance conflict that we and others have argued is the hallmark of anxiety (Gray & McNaughton, 2000; Bannerman *et al.*, 2004). On this basis one could argue that at the start of the black/white alley test the balance between approach and avoidance of the white section is firmly weighted in favour of avoidance, and hence anxiety levels would be lower at this point in the test than when the approach/avoidance conflict is more equally weighted. Alternatively, anxiety levels could be highest when the rat is physically located in the white section (i.e. the potentially more dangerous section) of the apparatus. In the present study, we found that HPC tissue O_2_ levels were significantly higher when rats were in the white vs. the black section but, on this result alone, we would not claim that the rats were necessarily more anxious in the white section. To reiterate, it is the approach/avoidance conflict that generates anxiety, not the specific stimuli themselves, and there is no way of knowing when the conflict is highest. Moreover, it is worth pointing out that these approach/avoidance conflict tests were designed as simple, rapid, single-trial paradigms capable of detecting anxiolytic drugs, and were not intended to delineate the time course of anxiety (Crawley & Goodwin, 1980; Shephard & Estall, 1984). Our rationale for using the black/white alley and food neophobia tests, as opposed to many other potentially anxiogenic tests, is that they are demonstrably more sensitive to vHPC than dHPC lesions (Bannerman *et al.*, 2002; McHugh *et al.*, 2004). The present study is the first to show that these vHPC lesion-sensitive tasks also elicit robust vHPC tissue O_2_ responses, thereby providing additional and convergent evidence for a preferential role for vHPC in anxiety.

### Physical activity and the dHPC

It might be argued that the dHPC tissue oxygen signals simply reflect the physical activity/movement of the animal. Of course any physical activity is going to result in an increase in heart rate and blood flow, which could, in turn, lead to an increase in tissue oxygen supply to the brain. One might expect that a systemic cardiovascular response would produce a global change in tissue oxygen levels and, as such, one might not expect region-specific changes, although it is equally possible that region-specific changes could result from local differences in the neuroanatomical organization of the cerebrovasculature. It is therefore important to include appropriate control groups in order to ascertain whether changes in tissue oxygen levels are due to physical activity rather than to information processing. To this end we included the one-arm control group in our spatial memory studies. These animals are matched for physical activity but have different memory demands during task performance, and are therefore the appropriate control group for this study. Thus, if the dHPC tissue O_2_ signal simply reflects the physical activity of the animal, then we would not see a difference between the eight-arm and one-arm groups during arm entries because their running speeds/activity were identical.

Although influenced by physical activity, our data clearly show that the dHPC tissue O_2_ signal is not simply a reflection of animal movement. We state this with confidence because there was a clear statistical difference between dHPC tissue O_2_ responses in the eight-arm and one-arm groups during arm entries, and yet running speeds were almost identical in the two groups. Thus, the difference between the eight-arm and one-arm groups is not explained by physical activity. Second, within the eight-arm and one-arm groups there were robust O_2_ signal differences between centre periods 1–3 and yet, again, the running speeds were virtually identical. Third, the simultaneous recordings of LFPs and tissue O_2_ during sleep and waking show unequivocally that the largest dHPC tissue O_2_ signals were actually recorded during REM sleep, in the absence of any animal movement. Moreover, these data suggest that dHPC tissue O_2_ signals have a close relationship with HPC theta activity, which is high during both physical movement and REM sleep. Previous studies have reported robust HPC single-unit activity during REM sleep (Poe *et al.*, 2000; Louie & Wilson, 2001), and the presence of theta activity, rather than movement or spatial processing *per se*, may be a prerequisite for spiking activity in the HPC during waking (Foster *et al.*, 1989; Czurko *et al.*, 1999).

### A preferential role for dHPC in spatial processing

During spatial information processing on the radial maze, dHPC tissue O_2_ signals showed a significant increase from baseline, in contrast to those in vHPC. This finding is consistent with a large body of evidence from human functional imaging, and lesion and electrophysiology studies in rodents. In humans, HPC activity during navigational tasks is centred on the posterior rather than the anterior region (Maguire *et al.*, 1997, 1998; Hartley *et al.*, 2003; Kumaran & Maguire, 2005). In rodents, dHPC but not vHPC lesions impair spatial memory in the watermaze, T-maze and radial arm maze (Moser *et al.*, 1995; Hock & Bunsey, 1998; Bannerman *et al.*, 1999; Pothuizen *et al.*, 2004). In addition, dHPC place cells are more numerous and have higher spatial resolution than those in vHPC (Jung *et al.*, 1994; Maurer *et al.*, 2005; Kjelstrup *et al.*, 2008). Collectively these data suggest a preferential role for dHPC in spatial processing.

Our radial maze experiments were based on a previous IEG study that found greater Fos expression in HPC following the eight-arm task compared with the one-arm task (Vann *et al.*, 2000). IEG expression offers high spatial resolution but, because it presents a single snapshot of cumulative neuronal activation occurring over many minutes or hours, it has no temporal resolution. Utilizing the high temporal resolution of CPA, our data extend those of Vann *et al.* by identifying ‘arm entries’ as the critical epoch responsible for the difference between the eight-arm and one-arm tasks. Importantly, this difference was not observed when animals crossed from the arms back into the central area, which was common to both tasks. In addition, this difference was not evident at the start of training but emerged later, and was driven by a reduction in tissue O_2_ signals in rats performing the one-arm task.

However, there is an apparent inconsistency in the data that requires further discussion – namely that whereas the dHPC O_2_ signal was strongly correlated with running speed in the black/white alley, running speed could not explain the dHPC O_2_ signal differences in the eight-arm and one-arm task during arm entries that were evident by the end of radial maze training. It is tempting to suggest that the dHPC tissue oxygen signal reflects the attention paid to the spatial cues and/or the amount of spatial information processing that occurs in the different experimental situations.

During the black/white alley test, as the trial proceeded and as the rat habituated to the novel environment, the increased familiarity of the spatial cues led to a reduction in exploration. This in turn led to a concomitant decrease in both the attention paid to the spatial cues and in running speed/activity. Thus, the tissue oxygen signal correlates highly with running speed because dHPC activity reflects the levels of exploration and the interest paid to the spatial cues. These decrease in parallel as the trial continues and the animal habituates to the environment.

In contrast, there is much less concordance between running speed/activity and the dHPC tissue O_2_ signal in well-trained animals performing the eight-arm and one-arm radial maze tasks. We have argued recently, based on studies in genetically modified mice, that rodents perform efficiently on win-shift, spatial working memory maze tasks by comparing the relative familiarity of the different arms of the maze (Sanderson *et al.*, 2010). Of course, training on the eight-arm task is likely to lead to directed or maintained attention towards the relative familiarity of the spatial cues. In contrast, rats in the one-arm condition are likely to reduce their levels of attention towards irrelevant spatial cues as training proceeds. Running speed/activity, which was equivalent in both groups, presumably reflects the motivation to run down the arms and collect rewards. Thus, under these conditions, running speed/activity will no longer reflect the level of attention that is paid to the spatial stimuli. In this regard, it is worth re-iterating that the eight-arm and one-arm groups only differed significantly from each other after extended training.

Consistent with the view that the dHPC signal is strongly influenced by the level of attentional processing that stimuli receive, previous studies have shown that the firing rate of dHPC units in rats is lower in familiar (habituated) than novel environments (Nitz & McNaughton, 2004; Csicsvari *et al.*, 2007; Cheng & Frank, 2008). Moreover, HPC units recorded in primates habituate to repeated presentation of the same visual stimulus (Rolls *et al.*, 1993). Interestingly, human neuroimaging studies have also shown that the HPC BOLD signal habituates to repeatedly presented stimuli (Yamaguchi *et al.*, 2004).

### What aspect of neuronal activity do tissue O_2_ signals represent?

There is considerable debate about what aspects of neuronal activity are reflected in the haemodynamic response (Attwell & Iadecola, 2002; Lauritzen, 2005; Logothetis, 2008). However, there is some agreement that the mechanisms controlling CBF (and hence increases in BOLD and tissue O_2_ signals) are driven primarily by afferent input to a region rather than spiking activity within it (Mathiesen *et al.*, 1998; Logothetis *et al.*, 2001), although in many cases these neuronal measures are likely to be correlated (Smith *et al.*, 2002). Consequently, perhaps the most consistent correlate of CBF has been aggregate measures of neuronal activity, such as the summed field potential (Mathiesen *et al.*, 1998; Masamoto *et al.*, 2008). Importantly, several studies have shown that increases in CBF can occur within 200–600 ms after stimulus onset (Silva *et al.*, 2000; Matsuura & Kanno, 2001; Norup Nielsen & Lauritzen, 2001), which justifies the fine temporal resolution used in our analyses. Moreover, haemodynamic responses do not reflect the metabolic need of the tissue (Powers *et al.*, 1996; Mintun *et al.*, 2001), but rather are principally controlled by glutamatergic neurotransmission (Faraci & Breese, 1993; Fergus & Lee, 1997; Lovick *et al.*, 1999). In this sense the coupling between neuronal activity and the haemodynamic response is best described as a feed-forward rather than feedback system (Attwell & Iadecola, 2002; Lauritzen, 2005). Therefore, tissue O_2_ responses are likely to index local glutamatergic neurotransmission.

In most cases increased neuronal activity leads to higher tissue O_2_ (or BOLD) signals, but some experiments have shown that highly localized neuronal activity can lead to decreased tissue O_2_ under some circumstances (Thompson *et al.*, 2003, 2005; Offenhauser *et al.*, 2005). This notwithstanding, in the present study we have interpreted increased tissue O_2_ as reflecting increased neuronal activity for the following reasons. First, studies showing tissue O_2_ decreases have all been in anaesthetized animals, and the brain regions under scrutiny have all had low basal activity punctuated by intense periods of stimulation. Tissue O_2_ signals represent a dynamic balance between consumption and supply, and in these experiments the parameters used may have maximized O_2_ consumption before CBF (and hence O_2_ supply) could compensate. In contrast, the present study recorded from the HPC of conscious rats and, like the BOLD signal in awake humans, under these circumstances the balance may favour O_2_ supply over consumption. Second, our combined recordings of LFPs and tissue O_2_ from dHPC showed that theta activity, a commonly used index of HPC activity, was highest in situations when tissue O_2_ was also highest (i.e. during REM sleep and active waking). Thus, in the present study the more parsimonious interpretation is that increases in the tissue O_2_ signal reflect increased neuronal activity.

### CPA as a translational technique

CPA-measured tissue O_2_ signals offer an analogue of BOLD fMRI primarily because both signals reflect the local increase in O_2_ delivery induced by increased neuronal activity. However, it is important to acknowledge the differences between the two techniques. Most obviously, fMRI is a whole-brain technique, whereas CPA requires the implantation of electrodes into discrete brain areas and therefore represents a regions-of-interest analysis. Second, CPA is invasive whereas fMRI is non-invasive. Third, the BOLD signal reflects changes in the ratio of oxy- to deoxyhaemoglobin, whereas CPA provides a direct and continuous measure of tissue O_2_ concentration. However, as both signals are similarly influenced by changes in local blood flow, blood volume and oxygen consumption, it is a reasonable assumption that changes in tissue O_2_ concentration are indicative of analogous changes in the BOLD signal (Zheng *et al.*, 2002; Offenhauser *et al.*, 2005; Thompson *et al.*, 2005; Viswanathan & Freeman, 2007). Indeed, we recently demonstrated a strong concordance between oxygen CPA signals and BOLD signals recorded simultaneously in the MRI scanner (Lowry *et al.*, 2010).

Moreover, CPA has several advantages over fMRI. First, it can be used in freely-moving animals, allowing it to be combined with invasive experimental manipulations (e.g. lesions, genetic interventions) that would not be possible in humans. Second, it has sub-second temporal resolution. Third, it can be combined with other co-localized measures such as unit activity or LFPs or other voltammetric techniques to measure neurotransmitter levels. Consequently, CPA offers a uniquely powerful method for investigating neuronal and O_2_-based signals simultaneously in behaving animals.

## Conclusions

In summary, this study used CPA to demonstrate a double dissociation of function in the HPC, with greater vHPC activity during anxiety and greater dHPC activity during spatial processing. Second, we demonstrate that dHPC activity is modulated by spatial memory demands. Finally, these data demonstrate that CPA has the potential to be a valuable translational technique between two of the largest areas of neuroscience – rodent behaviour and human functional imaging.

## Abbreviations

BOLD: blood oxygen level-dependent
CBF: cerebral blood flow
CPA: constant potential amperometry
CPE: carbon paste electrode
dHPC: dorsal hippocampus (hippocampal)
fMRI: functional magnetic resonance imaging
HPC: hippocampus (hippocampal)
IEG: immediate-early gene
ITI: inter-trial interval
LFP: local field potential
NREM: non-rapid eye movement
REM: rapid eye movement
vHPC: ventral hippocampus (hippocampal)

## Supporting Information

Fig. S1. *In vivo* validation of the tissue O_2_ signal.

Fig. S2. A flow diagram of the eight-arm working memory task on the radial maze.

Please note: As a service to our authors and readers, this journal provides supporting information supplied by the authors. Such materials are peer-reviewed and may be re-organized for online delivery, but are not copy-edited or typeset by Wiley-Blackwell. Technical support issues arising from supporting information (other than missing files) should be addressed to the authors.
